# 198. Comparison of Standard versus Extended Treatment Duration for Gram-negative Bloodstream Infections with Obstructing Nephrolithiasis or Urolithiasis

**DOI:** 10.1093/ofid/ofad500.271

**Published:** 2023-11-27

**Authors:** Tara L Harpenau, Adam C Clemens, Douglas A Brown

**Affiliations:** UC Health - West Chester Hospital, Cleves, Ohio; UC Health - West Chester Hospital, Cleves, Ohio; University or Cincinnati College of Medicine, Cincinnati, Ohio

## Abstract

**Background:**

There is minimal data regarding optimal duration of therapy in gram-negative bloodstream infections (GN-BSI) with obstructing nephrolithiasis/urolithiasis. Prescribing practices often include standard antibiotic duration (fixed duration with an antibiotic free period prior to stone management) or extended duration (continuous antibiotics until definitive stone management). This study compares outcomes between standard versus extended antibiotic durations in patients with GN-BSI with obstructing nephrolithiasis/urolithiasis.

**Methods:**

This IRB approved retrospective cohort study screened patients treated between 1/1/2016 and 1/1/2022. The standard duration group had an antibiotic free period of ≥ 7 days before definitive stone management and the extended duration group had continuous antibiotics without an antibiotic free period ≥ 7 days before definitive stone management. The primary outcome was infection recurrence before definitive stone management. Select secondary outcomes included revisit to care, antimicrobial resistance, and mortality. Continuous data utilized a t-test or Mann-Whitney U test and categorical data utilized either Chi-square or Fisher’s exact test.

**Results:**

After screening 405 encounters, there were 39 standard and 29 extended group patients. Median antibiotic duration was 16 (15,19) vs. 19 (16,23) days, p = 0.06 and time to definitive stone management was 52 (34,71) vs. 22 (15,25.5) days, p< 0.01 in the standard and extended group respectively. Infection recurrence was no different in the two groups, 5% (n=2) vs. 0%, p=0.5. Revisit to care was significantly higher in the standard group 28% (n=11) vs. 3% (n=1), p< 0.01, with the most common patient complaint of “concern for infection.”

**Conclusion:**

This study found no difference in infection recurrence between standard and extended antibiotic treatment strategies but was underpowered. A higher rate of revisit to care in patients who received standard courses of antibiotics is concerning and may also be influenced by a prolonged time to definitive stone management. Further data is necessary to determine the optimal antibiotic treatment strategy and optimal time of definitive stone management after GN-BSI with obstructing stones.

**Disclosures:**

**All Authors**: No reported disclosures

